# The ELIXIR channel in *F1000Research*

**DOI:** 10.12688/f1000research.7587.2

**Published:** 2016-05-04

**Authors:** Niklas Blomberg, Arlindo Oliveira, Barend Mons, Bengt Persson, Inge Jonassen

**Affiliations:** 1 ELIXIR Hub, Wellcome Genome Campus, Hinxton, Cambridgeshire, UK; 2 INESC-ID, Instituto Superior Técnico, Lisbon, Portugal; 3 Department of Human Genetics, Leiden University Medical Center, Leiden, Netherlands; 4 Department of Cell and Molecular Biology, Science for Life Laboratory, Uppsala University, Uppsala, Sweden; 5 Computational Biology Unit, Department of Informatics, University of Bergen, Bergen, Norway

**Keywords:** ELIXIR, ELIXIR Channel, bioinformatics

## Abstract

ELIXIR, the European life science infrastructure for biological information, is a unique initiative to consolidate Europe’s national centres, services, and core bioinformatics resources into a single, coordinated infrastructure. ELIXIR brings together Europe’s major life-science data archives and connects these with national bioinformatics infrastructures  - the ELIXIR Nodes. This editorial introduces the ELIXIR channel in *F1000Research*; the aim of the channel is to collect and present ELIXIR’s scientific and operational output, engage with the broad life science community and encourage discussion on proposed infrastructure solutions. Submissions will be assessed by the ELIXIR channel Advisory Board to ensure they are relevant to ELIXIR community, and subjected to *F1000Research* open peer review process.

## Introduction

Open data has long been the norm in molecular biology and genomics, and the major biomolecular archives and databases are accessed by thousands of scientists every day. As cost of data generation continues to plunge, ever-larger volumes of ever-more complex data must flow freely between platforms, laboratories and people. ELIXIR, the European Research Infrastructure for biological data, represents our collective efforts to coordinate data flows and sustain these vital data resources.

Europe has 500,000 life-scientists ^1^ working in thousands of laboratories across the continent. Managing the data needs in biology - from biomedicine to biodiversity - requires the coordination of local, national and international resources. ELIXIR brings together Europe’s major life-science data archives (including EMBL-EBI) and, for the first time, connects these with national bioinformatics infrastructures and resources. ELIXIR is a distributed infrastructure, based around the national bioinformatics infrastructures - our ELIXIR Nodes.

## Community engagement and adoption

ELIXIR is not building an infrastructure from scratch: Europe is the home of some of the world’s leading bioinformatics resources built by a strong and active community. Some European countries have had national bioinformatics organisations for many years - the SIB, Swiss Institute of Bioinformatics - for example has been operating since 1988. The challenge ahead of us is to further develop and embed community practices and standards and ensure that these are supported by reliable - and sustained - services that can form the basis of user’s daily workflows.

Addressing this challenge will require openness and transparency. ELIXIR will develop surveys, guidelines, technical reports, interface requirements and best practices to drive interoperability and the flow of data across Europe ^2^. Open publishing and peer review are ideal tools to ensure the community engagement that precedes adoption and embedding of standards into everyday workflows. We hope that this channel will highlight interesting approaches from all ELIXIR Nodes and participants and stimulate an active discussion on proposed infrastructure solutions.

## Scope of papers published in the channel

What is the scope of the channel? To put simply, it is ELIXIR. We welcome papers that describe ELIXIR services, technology developments, and architecture overviews as “Software Tool Articles, Method Articles or white papers”. ELIXIR Nodes and working groups are encouraged to publish surveys, best practice recommendations and workshop outcomes as “Meeting Reports” or “Opinion Articles”. And we welcome submissions that describe development and implementation of standards and collaborative efforts. ELIXIR projects will also publish reports on progress and deliverables through this channel.

## Advisory Board

The Advisory Board of the ELIXIR channel will streamline the publication process. The Board members - representatives of the ELIXIR Hub and the ELIXIR Nodes - will accept submissions based on the agreed scope for the channel and ensure that the articles published in the ELIXIR channels are relevant to ELIXIR community. However, once an article is accepted for submission to the channel we will not interfere in the open review process provided by *F1000Research*.

## Review process

Submissions to the ELIXIR Channel will first pass through the initial quality check by *F1000Research* editors and then forwarded to the ELIXIR Advisory Board. The Board will make sure that the article is relevant to ELIXIR and within its scope; if approved, the article will be published in the ELIXIR channel and opened for the *F1000Research* peer review.

A negative decision by the ELIXIR Advisory Board will preclude the paper’s publication in the ELIXIR channel, but not on the *F1000Research* platform as such. Authors will still be able to publish their manuscript outside the ELIXIR channel and subject it to the *F1000Research* open peer review.

## Conclusion

Open access and open source is central to the mission of ELIXIR. The ELIXIR channel in F1000Research platform will ensure that our outcomes, data and software are publicly accessible, without any limitation on their type or perceived impact. The open peer review system of *F1000Research* will provides an excellent platform to communicate broadly with the community and stimulate commentary and debate. We look forward to a lively discussion.
